# Restoring Colistin Sensitivity in Multidrug-Resistant Pathogenic *E. coli* Using Cinacalcet Hydrochloride

**DOI:** 10.3390/ijms252111574

**Published:** 2024-10-28

**Authors:** Chenchen Wang, Ziyi Zhang, Di Liu, Xiaodan Li, Zhaoran Zhang, Yan Zeng, Wenqi Dong, Chen Tan, Manli Liu

**Affiliations:** 1 Hubei Biopesticide Engineering Research Centre, Wuhan 430000, China; 2018302110164@webmail.hzau.edu.cn; 2 College of Veterinary Medicine, Huazhong Agricultural University, Wuhan 430000, China; zhangziyi@webmail.hzau.edu.cn (Z.Z.); 2024302110121@webmail.hzau.edu.cn (D.L.); xiaodanli@webmail.hzau.edu.cn (X.L.); zhangzhaoran@mail.hzau.edu.cn (Z.Z.); zengyanyan@webmail.hzau.edu.cn (Y.Z.); dongwq@mail.hzau.edu.cn (W.D.); tanchen@mail.hzau.edu.cn (C.T.)

**Keywords:** colistin, *mcr-1*, antibacterial, cinacalcet hydrochloride, combination therapy

## Abstract

Restoring colistin’s efficacy is crucial in addressing the resistance crisis of colistin. This study utilized a high-throughput screening method to identify 43 compounds from 800 FDA-approved drugs that exhibited significant antibacterial effects when combined with colistin. Among these, cinacalcet hydrochloride (CH) was selected for its potential synergistic effect with colistin against multidrug-resistant (MDR) *E. coli* strains, including *mcr-1*-positive strains. A series of experiments revealed that the combination of CH and colistin showed strong synergy, especially in *mcr-1*-positive strains, restoring colistin sensitivity. The combination significantly inhibited bacterial growth and reduced CFU counts more effectively than either drug alone. Additionally, CH and colistin together significantly inhibited biofilm formation and eradicated existing biofilms, as visualized through confocal microscopy. Mechanistic studies showed that the combination increased bacterial membrane permeability and disrupted membrane integrity. The treatment also elevated extracellular ATP release and ROS production, indicating oxidative stress-induced bacterial death. Safety evaluations showed that the combination did not increase toxicity in host cells. Finally, animal models further validated the combination’s efficacy. Overall, this study showed that the combination of colistin and CH significantly restored colistin sensitivity in *mcr-1*-positive *E. coli*, revealing their synergistic antibacterial mechanism involving membrane damage and oxidative stress, with promising clinical applications.

## 1. Introduction

Since the 1940s, the widespread use of antimicrobial drugs has dramatically improved the management of bacterial infections. However, the prolonged use of antibiotics has gradually exacerbated the problem of bacterial resistance, which has become a major global public health threat [1]. In recent years, the World Health Organization (WHO) has repeatedly warned that antibiotic resistance is one of the most severe challenges facing global health [2]. As resistant strains spread rapidly, many common infections have become increasingly challenging to treat, particularly those caused by multidrug-resistant (MDR) bacteria, which severely limit therapeutic options [3]. Among the various resistant pathogens, *Escherichia coli* (*E. coli*) poses a severe challenge due to its increasing resistance. *E. coli*, a common commensal bacterium found in the human and animal intestines, has evolved into a multidrug-resistant strain mainly due to the overuse of antibiotics in medical and agricultural fields [4,5]. In recent years, resistance in *E. coli* to commonly used antibiotics such as β-lactams, quinolones, and aminoglycosides has steadily increased [6,7,8]. According to data from the European Antimicrobial Resistance Surveillance Network (EARS-Net), the resistance rate of *E. coli* has reached nearly 50% in some countries. In parts of Asia and Africa, the rate is even higher [9]. This trend not only negatively affects patient outcomes but also increases the complexity of clinical treatments.

Even more concerning is the rapid emergence of colistin-resistant *E. coli*, which has become a global focal point of concern [7]. As one of the last-line defenses against Gram-negative bacteria, colistin has long been considered an effective treatment for infections caused by multidrug-resistant strains. However, the first report of the plasmid-mediated colistin resistance gene *mcr-1* in China in 2015 raised global alarm [10]. The horizontal transfer of the *mcr-1* gene via plasmids has significantly accelerated the spread of colistin resistance. The discovery of this gene marks a critical turning point in the fight against colistin-resistant *E. coli*, as its transmission has expanded beyond humans to animals and the environment, greatly complicating public health control efforts [11,12]. A study revealed that over 15% of 847 samples tested positive for *mcr-1*-carrying *E. coli*, and several human infection cases were reported [13]. This phenomenon indicates that colistin-resistant *E. coli* not only exists widely in animal husbandry but can also be transmitted to humans through the environment and the food chain, further exacerbating the global resistance crisis. Faced with this threat, restoring the clinical efficacy of colistin has become crucial. Restricting colistin use in agriculture and animal husbandry is an important measure to slow the spread of resistance genes [14]. In addition, in clinical settings, combination therapies, such as colistin combined with meropenem or tigecycline, have shown some efficacy in countering resistant strains [15]. Overall, the multidrug resistance of *E. coli*, especially colistin resistance associated with the mcr-1 gene, has become one of the core challenges in global antibiotic resistance. Thus, limiting the misuse of antibiotics and developing new antimicrobial therapies to restore colistin’s clinical efficacy is critical in addressing the colistin resistance crisis.

Screening FDA-approved drug libraries has become one of the most effective approaches to identifying potential antibiotic adjuvants [16]. The FDA drug library includes many drugs that have already undergone extensive clinical testing, with proven safety and efficacy profiles. High-throughput screening of this drug library allows for the rapid identification of molecules with potential adjuvant properties. This approach not only accelerates the clinical translation of drugs but also reduces concerns about drug toxicity and safety [17]. Several drugs initially used for cancer, antiviral, and anti-inflammatory treatments have been found to inhibit bacterial resistance mechanisms during screening, demonstrating excellent antibacterial effects when combined with antibiotics [16,18,19,20]. These studies indicate that antibiotic adjuvant development holds significant potential, particularly in addressing the growing threat of resistant bacteria. Therefore, screening for potential colistin adjuvants using the FDA-approved drug library has become an essential strategy for extending the clinical use of colistin and offers promising therapeutic options in combating resistant bacterial infections. Whole-cell inhibition-based high-throughput screening enables the rapid evaluation of thousands of compounds to identify adjuvants that can synergize with colistin to counteract resistant bacteria [21]. This approach not only accelerates the discovery of colistin adjuvants but also provides new therapeutic options for colistin in treating multidrug-resistant infections. In this context, the FDA-approved drug library was an essential resource for adjuvant screening. The library includes thousands of drugs that have been validated for safety and efficacy [17]. Screening these drugs can rapidly identify molecules capable of restoring colistin sensitivity.

In this study, we utilized the FDA-approved drug library for high-throughput whole-cell inhibition screening of colistin adjuvants. The results revealed that cinacalcet hydrochloride (CH), an FDA-approved drug, exhibited significant potential as a colistin adjuvant. CH was initially used to regulate calcium receptors without drug-induced liver injury concern [22,23]. CH enhanced colistin’s bactericidal effect against multidrug-resistant Gram-negative bacteria, restoring colistin sensitivity, particularly in combating *mcr-1*-positive strains, which made CH a powerful drug for restoring colistin sensitivity. CH combination with colistin offers a new therapeutic approach for treating multidrug-resistant bacterial infections. Moreover, the results demonstrated that the FDA-approved drug library could serve as an essential source for discovering antibiotic adjuvants, providing a practical solution to the global antibiotic resistance crisis.

## 2. Results

### 2.1. High-Throughput Whole-Cell Inhibition Screening Identified Cinacalcet Hydrochloride as a Colistin Adjuvant

**Figure 1:**
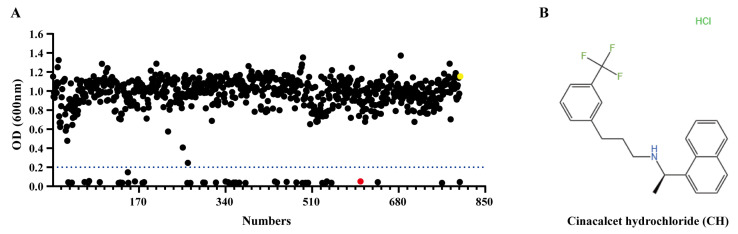
High-throughput whole-cell inhibition screening for synergistic colistin antimicrobials. (**A**) The screening drug concentration was 25 μM in combination with 0.5 μg/mL colistin E (one-eighth of the lowest inhibitory concentration, COL). Strain selection was *mcr-1*-positive MDR *E. coli* 42. Red dots represented CH and yellow dots represented bacterial control; (**B**) Chemical structural formula of CH.

High-throughput whole-cell antibacterial screening technology is a rapid and effective method for drug screening, offering significant advantages. This method can quickly identify candidate drugs with synergistic antibacterial effects from large compound libraries, saving time and costs while ensuring the clinical relevance of the screening results by detecting antibacterial activity at the whole-cell level [21]. Therefore, in this study, we used a high-throughput whole-cell antibacterial screening method to search for potential drugs that could synergize with colistin from 800 FDA-approved drugs. As shown in Figure 1A, among the 800 drugs initially screened, 43 drugs demonstrated significant antibacterial effects when combined with colistin (OD600 < 0.2). After excluding some conventional antibiotics and previously reported drugs, we randomly selected four drugs that exhibited antibacterial effects when combined with colistin and three drugs that did not, to test the feasibility of the screening method. As shown in Table 1, under consistent concentration conditions, the four effective drugs in the initial screening also restored colistin sensitivity in multidrug-resistant bacteria. In comparison, the three ineffective drugs did not, which demonstrated the reliability of the screening method used in this study. Finally, we selected cinacalcet hydrochloride (CH), which showed the best synergistic effect among the four drugs, for further study. The structural formula is shown in Figure 1B.

### 2.2. Synergistic Antibacterial Effect of CH and COL on Multidrug-Resistant E. coli

To further evaluate the synergistic antibacterial effect of cinacalcet hydrochloride and colistin, we performed MIC tests on clinical MDR *E. coli* isolates. As shown in Table S1, these clinical isolates were all multidrug-resistant strains, and six of them were *mcr*-1-positive (Figure S1). Except for the MIC value of CH against the standard *E. coli* strain ATCC25922, which was 32 µg/mL, the MIC values of CH against the remaining clinical strains ranged from 64 to 256 µg/mL, and for some strains, the MIC value exceeded 256 µg/mL, indicating weak or no antibacterial activity of CH on its own (Table S1). Next, this study randomly selected three *mcr-1*-positive and three *mcr-1*-negative strains for checkerboard analysis to test the combined effect of CH and colistin E. The results showed that the combination of colistin E and cinacalcet hydrochloride exhibited synergy in all strains, with FICI values below 0.5.

**Figure 2:**
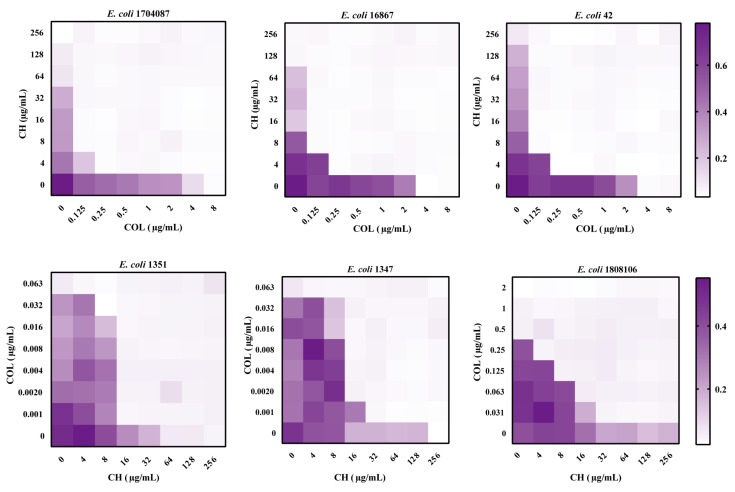
Checkerboard analysis of the synergistic antimicrobial effect of colistin and CH on both colistin-resistant and non-colistin-resistant *E. coli*. *E. coli* 1704087, *E. coli* 16867, and *E. coli* 42 were *mcr-1*-positive strains. *E. coli* 1351, *E. coli* 1347, and *E. coli* 1808106 were *mcr*-1 negative strains.

Specifically, the FICI values for the three *mcr-1*-positive strains, *E. coli* 1704087, *E. coli* 16867, and *E. coli* 42, were 0.047, 0.094, and 0.063, respectively (Figure 2). Similarly, the FICI values for the three *mcr-1* negative strains, *E. coli* 1351, *E. coli* 1347, and *E. coli* 1808106, were 0.078, 0.094, and 0.094, respectively (Figure 2). This confirmed the synergistic effect of CH and colistin E in both colistin-resistant and non-colistin-resistant strains. Overall, the data further demonstrated that CH not only restored colistin E sensitivity in colistin-resistant strains but also enhanced the antibacterial activity of colistin in colistin-sensitive strains.

### 2.3. Bactericidal Effect of CH and COL on mcr-1-Positive MDR E. coli

**Figure 3:**
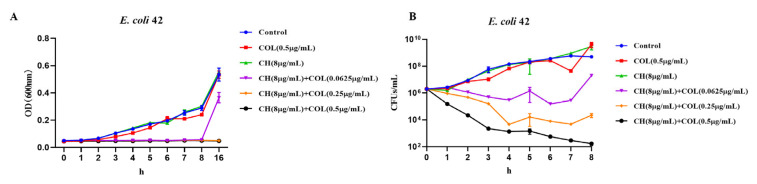
Antibacterial effect of CH combined with colistin against *mcr-1*-positive *E. coli* 42. (**A**) Growth curve assay of *E. coli* 42; (**B**) Time-kill assay of *E. coli* 42. Data were mean ± SEM for *n* = 3 biologically independent experiments.

Colistin, as a peptide antibiotic, has been used as a last line of defense in treating MDR Gram-negative bacterial infections. However, the emergence of *mcr-1* has significantly reduced the effectiveness of colistin, limiting its clinical use in treating resistant infections [10]. Therefore, subsequent experiments focused on evaluating the bactericidal activity of CH and colistin in *mcr-1*-positive MDR *E. coli*. The growth curve of *mcr-1*-positive MDR *E. coli* 42 (Figure 3A) showed that bacterial OD600 values increased over time in both the control group and in groups treated with either colistin (0.5 μg/mL) or CH (8 μg/mL). However, bacterial growth was significantly inhibited when CH and colistin were used together. In the group treated with CH (8 μg/mL) and colistin (0.0625 μg/mL), OD600 values remained unchanged for the first 8 h but increased slightly afterward, although still lower than in the control and single-drug groups (Figure 3A). In the groups treated with CH (8 μg/mL) and colistin (0.5 μg/mL or 0.25 μg/mL), OD600 values remained nearly constant, indicating potent inhibition of bacterial growth (Figure 3A). Subsequently, time-kill curves were used to assess bacterial CFU counts over time (0–8 h) in different treatment combinations. Results showed that bacterial CFU counts remained high in the control group and groups treated with either colistin (0.5 μg/mL) or CH (8 μg/mL), indicating normal bacterial proliferation (Figure 3B). However, the combination treatment of CH and colistin significantly reduced bacterial CFU counts, with the most pronounced bactericidal effect observed in the groups treated with CH (8 μg/mL) and colistin (0.5 μg/mL), where CFU counts rapidly decreased to 10^2^ CFU/mL within 8 h (Figure 3B). However, whether the bactericidal effect of the combination of CH and colistin improved with increasing CH concentration still required further experimental validation to explore the optimal ratio of the two agents. Overall, these data demonstrated the significant synergistic antibacterial effect of CH and colistin in *mcr-1*-positive MDR *E. coli*.

### 2.4. The Effect of CH Combined with COL on Biofilm Formation and Eradication in mcr-1-Positive MDR E. coli

**Figure 4:**
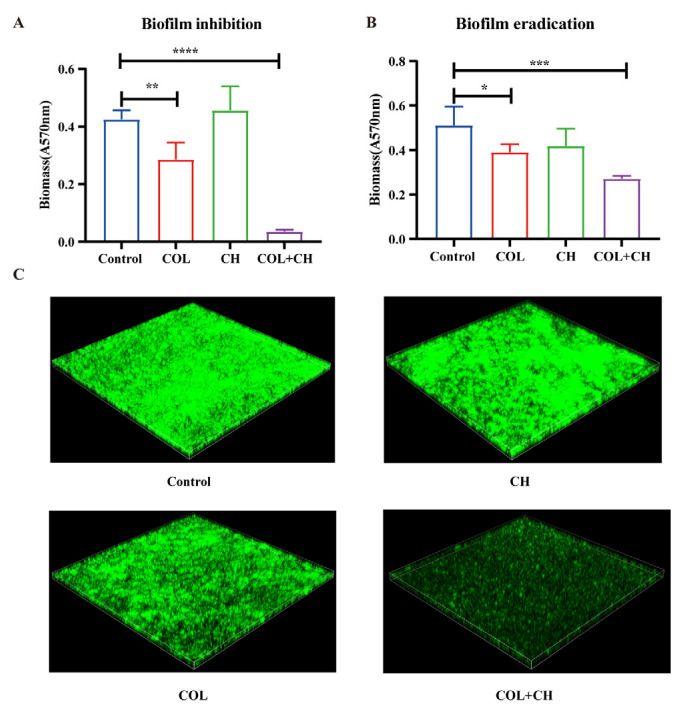
Effects of CH combined with colistin on the biofilm of *mcr-1*-positive *E. coli* 42. (**A**) Inhibition of biofilm formation assay. Colistin (2 μg/mL) or CH (8 μg/mL) alone, CH (8 μg/mL) combined with colistin (2 μg/mL); (**B**) Eradication of biofilm assay. Data were mean ± SEM for *n* = 5 biologically independent experiments. * *p* < 0.05, ** *p* < 0.01, *** *p* < 0.001, **** *p* < 0.0001 vs. control; (**C**) Staining of bacterial mature biofilm by SYTO 9 (5 μM).

Biofilm formation in *E. coli* is an important survival strategy in adverse environments. Biofilms not only allow bacteria to colonize host tissues but also enhance their resistance to antibiotics and the host immune system [24]. This study further evaluated the inhibitory and eradication effects of CH and colistin on bacterial biofilm in *mcr-1*-positive *E. coli*. In the biofilm inhibition assay, colistin (2 μg/mL) alone was able to reduce biofilm formation, but the effects were limited (Figure 4A). Biofilm mass in the colistin group was significantly lower than in the control group (*p* < 0.01), while there was no significant difference between the CH group and the control (Figure 4A). However, when CH and colistin were used together, biofilm mass was significantly reduced and almost completely inhibited (*p* < 0.0001), indicating an apparent synergistic inhibitory effect (Figure 4A). In the biofilm eradication assay, colistin alone showed limited efficacy in clearing established biofilms, with the impact being weaker than the control (*p* < 0.05) (Figure 4B). However, the combination of CH and colistin significantly enhanced biofilm eradication, and the residual biofilm mass was markedly reduced compared to the control group (*p* < 0.001), demonstrating a strong synergistic clearing effect (Figure 4B). Furthermore, confocal laser scanning microscopy images revealed the impact of different treatments on biofilm structure. The biofilms in the control group and those treated with either colistin or CH alone were thick and covered most of the visual field (Figure 4C). In contrast, after the combined treatment, the intensity of the green fluorescence signal from live bacteria was significantly reduced. The density and thickness of the biofilm were markedly decreased, indicating a substantial reduction in the number of live bacteria within the biofilm (Figure 4C). Both the data and imaging results consistently showed that the combination of colistin and CH had a significant effect on biofilm inhibition and eradication.

### 2.5. CH and Colistin Combination Caused Bacterial Membrane Damage

**Figure 5:**
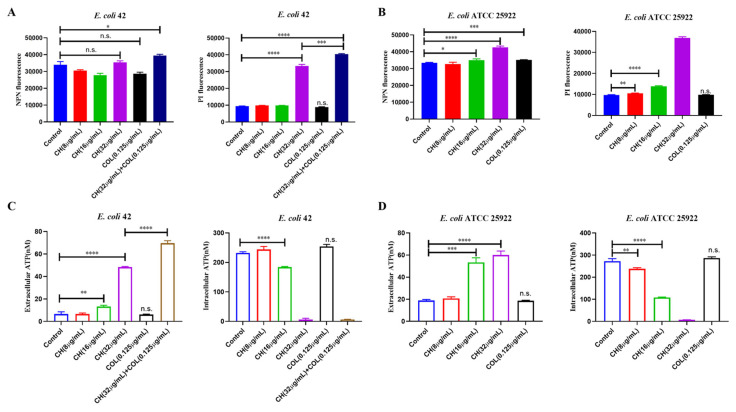
The combination of CH and colistin disrupted bacterial membrane integrity. NPN or PI fluorescence intensity detection after treatment of *E. coli* 42 (**A**) or *E. coli* ATCC 25922 (**B**) with drugs; The measurement of extracellular and intracellular ATP after treatment of *E. coli* 42 (**C**) or *E. coli* ATCC 25922 (**D**) with drugs. Data were mean ± SEM for *n* = 3 biologically independent experiments. n.s. represents no significance, * *p* < 0.05, ** *p* < 0.01, *** *p* < 0.001, **** *p* < 0.0001 vs. control.

To explore the mechanism underlying the synergistic antibacterial effect of CH and colistin, this study examined membrane permeability, intracellular and extracellular adenosine triphosphate (ATP) changes, reactive oxygen species (ROS), and nitric oxide (NO) production in *E. coli*. N-Phenyl-1-naphthylamine (NPN) fluorescence was used to assess outer membrane permeability, as NPN enters cells and emits fluorescence only when the membrane is damaged. For the *mcr-1*-positive MDR *E. coli* 42, NPN fluorescence revealed no significant changes in outer membrane permeability when CH was used at concentrations of 8–32 μg/mL (Figure 5A). Similarly, colistin alone (0.125 μg/mL) did not significantly alter NPN fluorescence (Figure 5A). However, the combination of CH (32 μg/mL) and colistin (0.125 μg/mL) significantly increased NPN fluorescence, with the highest fluorescence observed, indicating a synergistic effect in disrupting the outer membrane (Figure 5A). Similarly, Propidium Iodide (PI) fluorescence was used to assess changes in inner membrane permeability. The combination of CH and colistin in MDR *E. coli* 42 also significantly increased PI fluorescence, indicating increased inner membrane permeability and further demonstrating that the bacterial membrane integrity was compromised (Figure 5A). In contrast, neither CH (8–16 μg/mL) nor colistin (0.125 μg/mL) alone had a significant effect on PI fluorescence (Figure 5A). However, when CH was used alone at 32 μg/mL, a significant increase in PI fluorescence was observed (Figure 5A). To further explore the effect of CH when used alone, we examined its impact on the membrane of the standard *E. coli* strain ATCC25922. As shown in Figure 5B, NPN fluorescence revealed that CH started to affect the outer membrane at a concentration of 16 μg/mL. In contrast, PI fluorescence showed that CH affected the inner membrane at 8 μg/mL, indicating that CH alone could increase membrane permeability. Additionally, ATP leakage assays were performed to further assess the impact of the CH and colistin combination on bacterial membrane integrity. In both *E. coli* 42 and *E. coli* ATCC25922, the combination of CH and colistin resulted in the highest levels of extracellular ATP (*p* < 0.0001), consistent with increased membrane permeability. Meanwhile, the lowest levels of intracellular ATP were observed in the combination treatment group (*p* < 0.0001) (Figure 5C). Furthermore, ROS assays revealed that the combination of CH and colistin significantly increased ROS production in both *E. coli* 42 and *E. coli* ATCC25922, with fluorescence signals reaching their highest values (*p* < 0.0001) (Figure S2A,B). However, when measuring NO production, no significant changes were observed, suggesting that the combination of CH and colistin does not affect NO levels under the experimental conditions, and the mechanism may not be related to nitrogen stress (Figure S2C,D). These data suggested that the combination of CH and colistin induced strong oxidative stress and disrupted the bacterial membrane structure, ultimately leading to bacterial death.

### 2.6. Safety Evaluation of CH and COL Combination Therapy

**Figure 6:**
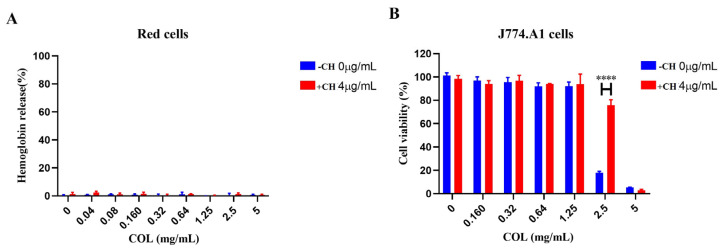
Toxic effects of CH combined with colistin on red blood cells (**A**) or J774.A1 macrophage cells (**B**). Data were mean ± SEM for *n* = 3 biologically independent experiments. **** *p* < 0.0001.

The safety of colistin use is significantly limited by its nephrotoxicity and neurotoxicity, especially at high doses or with prolonged use, which reduces its therapeutic efficacy [25]. To assess whether CH affected the toxicity of colistin, we analyzed the hemolysis and cytotoxicity of colistin in the presence of CH. The hemolysis assay of the drug combination showed that as the concentration of colistin increased, the hemolysis rate of red blood cells treated with the drug combination (red, 4 μg/mL) remained at low levels, almost close to 0%, compared to red blood cells untreated with CH (blue, 0 μg/mL) (Figure 6A). This indicates that the addition of CH did not increase the hemolytic effect of colistin. Figure 6B shows the effect of colistin alone and in combination with CH on the viability of J774.A1 macrophage cells. As the concentration of colistin increased, cell viability significantly decreased. At a colistin concentration of 2.5 mg/mL, the viability of cells dropped to about 20%, while cells in the presence of CH dropped to close to 80%, with this difference being statistically significant (****, *p* < 0.0001). At the highest concentration (5 mg/mL), cell viability in all treatment groups dropped to nearly 0%, indicating high toxicity of colistin at this concentration, causing almost complete cell death. However, adding CH did not increase the toxicity of colistin towards eukaryotic cells. These data indicated the safety of combining CH with colistin.

### 2.7. Protective Effect of CH and COL Combination Therapy in Animal Models of mcr-1-Positive Bacterial Infection

**Figure 7:**
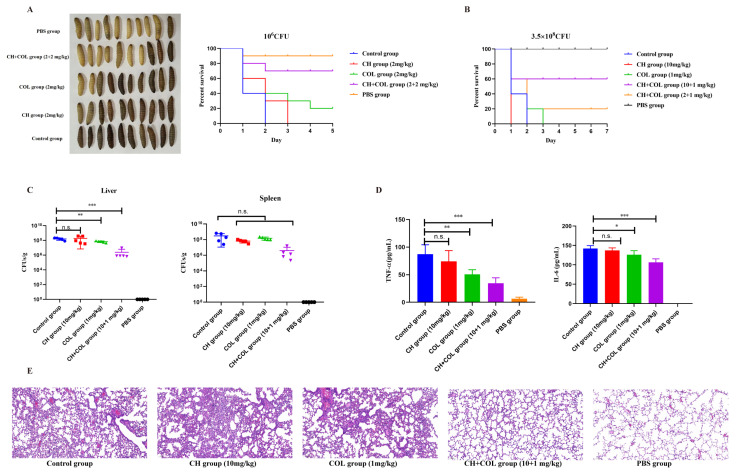
Therapeutic efficacy of CH combined with colistin in animal infection models. (**A**) Protection rates of drugs in the *Galleria mellonella* larva infection model; (**B**) Protection rates of drugs in the mouse acute lethal sepsis model; Therapeutic effect of CH combined with colistin in non-lethal mouse infection model. Bacterial load in liver and spleen (**C**), levels of TNF-α and IL-6 in blood serum (**D**), histopathologic changes (**E**) in the lungs of infected mice after drug treatment. Data were mean ± SEM. (n.s. represents no significance, * *p* < 0.05, ** *p* < 0.01, *** *p* < 0.001).

To further understand the therapeutic potential of combining CH with colistin in treating *mcr-1*-positive bacterial infections, animal experiments were conducted using animal models infected with *mcr-1*-positive *E. coli* 42. In the *Galleria mellonella* larva bacterial infection model, the appearance of larvae in different treatment groups was observed after infection with 10⁶ CFU of bacteria, and their survival was recorded. The larvae in the control group and single-drug treatment groups appeared dull, showing severe lesions and damage, whereas the larvae in the CH + COL group (2 + 2 mg/kg) exhibited significantly less infection damage (Figure 7A). In the survival statistics, all larvae in the control group died within 2 days post-infection, while those in the CH group all died within 3 days. The survival rate in the COL group was also low, with only 20% of larvae surviving (Figure 7A). In contrast, the survival rate in the CH + COL group significantly increased, with about 70% of the larvae still alive on day 4 (Figure 7A), demonstrating a significant protective effect of the combination therapy in the wax moth model. Similarly, in the mouse acute lethal sepsis model, all mice in the control group and single-drug treatment groups died within 3 days of infection (Figure 7B). However, the CH + COL groups with two different doses (2 + 2 mg/kg and 10 + 2 mg/kg) improved the survival rate of the infected mice, increasing survival to 20% and 60%, respectively (Figure 7B). Next, a sublethal dose of 1 × 10^8^ bacteria was used to evaluate the therapeutic effect of CH and colistin in infected mice. After 12 h of treatment, the bacterial load in the organs, tissue pathology, and blood levels of inflammatory cytokines were measured. Compared to the untreated group, both the colistin and combination groups showed a significant reduction in bacterial load in the liver, with an average decrease of 0.38 and 1.81 log units, respectively (Figure 7C). In the spleen, although there was no statistically significant difference between the combination group and the control group, the combination group had the most significant reduction in bacterial load compared to the other groups (Figure 7C). In the serum levels of TNF-α and IL-6, the CH + COL group (10 + 1 mg/kg) had significantly lower levels than the control group, with the combination group exhibiting the lowest levels of inflammatory cytokines, indicating that the combination therapy effectively suppressed the release of inflammatory factors (Figure 7D). Finally, in the pathological examination of lung tissue from the different groups, the control group and single-drug treatment groups showed severe lung tissue damage, including significant hemorrhage and inflammatory cell infiltration (Figure 7E). In contrast, the CH + COL group (10 + 1 mg/kg) displayed relatively intact lung tissue structure, reduced inflammatory infiltration, and significantly improved pathology (Figure 7E). These experiments demonstrated that the effect of combination therapy was significantly better than using colistin or CH alone, suggesting that this combination strategy held potential clinical application value for treating infections caused by *mcr-1*-positive bacteria.

## 3. Discussion

The discovery of the plasmid-mediated colistin resistance gene *mcr-1*, particularly its widespread dissemination in *E. coli*, has severely compromised colistin’s clinical efficacy [10]. Exploring novel antimicrobial strategies to restore colistin’s clinical effectiveness has become a key focus in infection treatment research. This study aimed to utilize a high-throughput screening method to identify drugs that could restore colistin’s antibacterial activity, focusing on the combination of CH and colistin against *mcr-1*-positive multidrug-resistant *E. coli*. Through a series of in vitro and in vivo experiments, the research found that the combination of CH and colistin exhibited a significant synergistic effect in *mcr-1*-positive strains. This not only provides a new therapeutic strategy for restoring colistin’s antibacterial efficacy but also offers novel insights and approaches for combating the growing crisis of multidrug resistance.

Several studies have explored the use of combination therapies to enhance colistin’s antibacterial effects and overcome resistance. Tacão et al. (2017) demonstrated that the combination of colistin and meropenem showed significant synergy against multidrug-resistant Gram-negative bacteria, particularly in inhibiting *mcr-1*-positive strains [26]. Similarly, Zhou et al. (2020) further confirmed that combining colistin with tigecycline enhanced its antibacterial activity against multidrug-resistant bacteria [27]. In contrast, this study innovatively introduced the FDA-approved compound CH as a potential adjuvant for colistin. The results showed that the combination of CH and colistin exhibited significant synergy in *mcr-1*-positive *E. coli*. Notably, unlike antibiotics such as meropenem and tigecycline, CH did not have direct antibacterial activity [22]. In MIC tests against multidrug-resistant *E. coli*, CH had MIC values ranging from 64 to 256 µg/mL, indicating weak or no antibacterial effect when used alone. However, when combined with colistin, its synergistic effect significantly enhanced colistin’s antibacterial activity. This finding was more valuable than existing antibiotic combination strategies, as CH, a non-antibiotic drug [22,28], can reduce the required antibiotic dosage, thus minimizing the risk of resistance development. Similarly, the FDA-approved non-antibiotic drug melatonin has likewise proven to be a promising colistin adjuvant against *mcr-1*-positive Gram-negative pathogens [20]. This study is the first to demonstrate the advantages of CH in combination therapy, showing that it not only enhances colistin’s antibacterial potency but also allows for a reduced colistin dosage and exhibits high safety. This also suggests that non-antibiotic drugs hold significant potential in restoring the efficacy of traditional antibiotics.

The combination of colistin and CH demonstrated significant synergistic antibacterial effects both in vitro and in vivo, which aligned with findings from Wang et al. regarding enhanced antibiotic efficacy through combination therapy [29,30]. Although compared to the FICI index, there was little difference in the effectiveness of CH in combination with colistin against colistin-resistant and susceptible strains. However, it seemed that the combination of CH and colistin was more effective against colistin-resistant strains than colistin-sensitive strains from the heat map displayed by the checkerboard method. This also differed from some reports in the references [31,32,33]. Here, we hypothesize that other mechanisms of action still exist for the synergistic antimicrobial action of CH and colistin, which need to be further explored. Additionally, this study found that the combination of colistin and CH not only inhibited biofilm formation but also significantly eradicated pre-formed biofilms, offering new possibilities for treating biofilm-associated infections. Previous studies have shown the potential of antibiotic combination strategies in treating biofilm infections [34]. The results of this study further support this notion and provide evidence for a novel drug combination. To investigate the mechanism of synergy between colistin and CH, this study further analyzed their effects on bacterial membrane permeability, intracellular and extracellular ATP levels, and ROS production. The combination of CH and colistin significantly increased ROS production in bacteria, suggesting that oxidative stress plays a key role in their bactericidal mechanism. This finding is consistent with the study by Kohanski et al. (2007), which highlighted the critical role of oxidative stress in bacterial death [35]. However, NO production did not show significant changes under the experimental conditions, suggesting that the combination had a limited effect on bacterial nitrogen stress responses, providing directions for future studies on other bacterial stress responses.

The clinical use of colistin is limited by its nephrotoxicity and neurotoxicity [36]. CH is an orally active, allosteric agonist of Ca receptor (CaR) and is used for cardiovascular disease treatment and parathyroid hyperplasia [22,28]. This study initially evaluated the safety of the CH and colistin combination in cell-based experiments, finding that the combination did not increase hemolysis of red blood cells or significantly increase macrophage cytotoxicity. On the contrary, the addition of CH reduced colistin’s cytotoxicity in some cases, suggesting that CH may have the potential to mitigate colistin’s toxicity. This finding offers a new approach to reducing the adverse effects of colistin in future clinical applications. Furthermore, the results from animal experiments support the clinical potential of combination therapy, demonstrating the potential clinical value of this combination strategy. This was also evident in previous studies reporting that combination therapy showed great therapeutic potential, whether the treatment was administered intraperitoneally or by gavage [31,32,37]. Despite the significant findings, there are limitations to this study. The research primarily focused on in vitro experiments and animal models, and the safety and efficacy of the CH and colistin combination have yet to be validated in larger clinical trials. Additionally, the study did not explore the potential of CH and colistin in other resistant bacterial strains. Future studies could expand to other multidrug-resistant pathogens to investigate the broader antibacterial applicability of this combination.

## 4. Materials and Methods

### 4.1. Bacterial Strains, Growth Conditions, and Drugs

*E. coli* ATCC25922 was obtained from the American Type Culture Collection (ATCC). Clinical isolates of *E. coli* (1240, 1351, 17039, 1347, 1808106, 19097, 17039, 1244, 1704086, and 42) were isolated from porcine clinical samples and identified and stored by our laboratory. Defibrinated sheep red blood cells were purchased from Yuan Ye Bio. Bacterial strains were cultured according to Clinical and Laboratory Standards Institute (CLSI) guidelines. Lyophilized bacteria stored in the laboratory were inoculated using a loop to transfer a small amount of the lyophilized powder to an LA plate and incubated overnight at 37 °C. Single colonies were then picked and transferred to LB broth and cultured overnight at 37 °C in a shaking incubator at 180 r/min. Mouse macrophage J774.A1 cells were preserved, expanded, and passaged in our laboratory. The cells were seeded in freshly preheated Dulbecco’s modified Eagle’s medium (DMEM) (Gibco, Gibco, Grand Island, NY, USA) medium (containing 10% FBS, 100 units/mL penicillin/streptomycin) and transferred to culture flasks in a 5% CO_2_ incubator. Cinacalcet hydrochloride and the FDA compound library were purchased from Topscience (Shanghai, China). DMSO (Sigma-Aldrich, St. Louis, MO, USA) was used to dissolve the drugs in this study.

### 4.2. Screening Protocol and MIC Determination

Drugs were randomly selected from a library of FDA drugs collected by Topscience. A total of 800 FDA-approved compounds were collected (Table S2), each at a concentration of 25 μM, in combination with 0.5 μg/mL colistin (one-eighth of its MIC). The *mcr-1*-positive *E. coli* 42 was used. In brief, colistin and the drugs were diluted in Mueller–Hinton broth (MHB). The drug dilutions were mixed with equal amounts of bacterial suspension in sterile 96-well plates (Corning Costar^®^ 3599, Corning, NY, USA) and incubated at 37 °C for 24 h. Absorbance at 600 nm was measured using a microplate reader. Pure MHB served as the negative control, and bacterial samples served as the positive control. The MIC values of drugs were determined using the standard broth microdilution method according to CLSI guidelines [38]. The test drugs were first dissolved to make a stock solution at 1000 μg/mL, and working solutions were prepared by diluting the stock in MHB. The working solutions were then further diluted in a twofold series in 96-well plates (final volume 100 μL). Bacteria were washed three times with PBS and adjusted to a final concentration of 2 × 10^6^ CFU/mL. 100 μL of bacterial suspension was added to each well containing the drug solution, and the 96-well plates were incubated at 37 °C for 18 h. The MIC was defined as the lowest concentration of the drug that completely inhibited visible bacterial growth. Each drug was set up with three biological replicates.

### 4.3. Identification of mcr-1Positive Strains

The *mcr-1* gene sequence (Gene ID: KY271416.1) was obtained from the NCBI database. Primers were designed using Primer Premier 5.0 and SnapGene Viewer software 7.2 and synthesized by Genscript Biotech (Wuhan, China). The following primers were used: *mcr-1*-F: ATGATGCAGCATACTTCTGTGTGGTA; *mcr-1*-R: TCAGCGGATGAATGCGGTGCGG. The *mcr-1* gene was amplified from clinical isolate genomic DNA using PCR. The PCR reaction was set up as follows: 95 °C for 3 min; 95 °C for 15 s, 58 °C for 15 s, 72 °C for 30 s, for 30 cycles; and a final extension at 72 °C for 5 min. *E. coli* ATCC25922 was used as a negative control.

### 4.4. Checkerboard Study

The FICI (Fractional Inhibitory Concentration Index) was calculated using the checkerboard method. Colistin and CH were diluted along the rows and columns of a 96-well plate. Bacteria cultured overnight were adjusted to an OD600 of approximately 0.5, washed with PBS, and adjusted to 2 × 10⁶ CFU/mL in MHB. 100 μL of MHB was added to each drug-containing well of the 96-well plate. The plates were incubated at 37 °C for 18 h. The MIC was recorded as the lowest drug concentration where no visible growth was observed. FICI was calculated using the formula: FICI = (MIC of drug A in combination/MIC of drug A alone) + (MIC of drug B in combination/MIC of drug B alone). FICI values were interpreted as follows: FICI ≤ 0.5 indicates synergy; 0.5 < FICI ≤ 1 indicates additivity; 1 < FICI < 2 indicates no interaction (or indifference); FICI ≥ 2 indicates antagonism [39]. Each group was set up with three biological replicates.

### 4.5. Growth Curve Measurement

*E. coli* 42 was cultured overnight in liquid medium at 37 °C and 180 rpm in a shaking incubator. The culture was then diluted 1:100 into MH medium and grown until the OD600 reached 0.6. The bacteria were centrifuged, resuspended in PBS buffer, and adjusted to a concentration of 2 × 10⁶ CFU/mL for later use. The drugs were prepared at the appropriate concentrations, then mixed 1:1 with the bacterial suspension in 2 mL EP tubes. The tubes were incubated at 37 °C in a shaking incubator at 200 rpm. The effect of the drugs on bacterial growth was assessed by measuring absorbance at 600 nm every hour [37]. Each group was set up with three biological replicates.

### 4.6. Time-Kill Curve Measurement Analysis

*E. coli* 42 was collected during the logarithmic growth phase, washed three times with PBS, centrifuged, and resuspended in PBS buffer to a final concentration of 1 × 10⁶ CFU/mL for later use. The untreated group served as the control, and the appropriate concentrations of CH and colistin were added to the other groups. Every hour, 100 μL of the bacteria-drug mixture was taken and serially diluted 10-fold for bacterial counting. Measurements were taken over 8 h, and a kill curve was plotted [40]. Each group was set up with three biological replicates.

### 4.7. Biofilm Formation Inhibition and Biofilm Eradication Assay

The experimental procedures refer to the previous references with minor modifications [41,42]. Clinical multidrug-resistant *E. coli* 42 was inoculated in LB broth and shaken at 37 °C overnight. The following day, the culture was diluted to 0.5 McFarland standard (1.5 × 10⁸ CFU/mL) and then further diluted 1:100 to a final concentration of 1.5 × 10⁶ CFU/mL for the biofilm formation assay. Based on MIC data, appropriate concentrations were set for the combination treatment group (colistin + CH, 2 μg/mL + 8 μg/mL), the single-drug control groups (colistin alone, 2 μg/mL; CH alone, 8 μg/mL), and the untreated control group. In a 96-well plate, 100 µL of the bacterial suspension was added to each well, followed by 100 µL of the drug solution to achieve the desired final concentrations. The plates were incubated at 37 °C for 24 h to allow biofilm formation. After incubation, each well was carefully washed three times with PBS to remove non-adherent cells. Then, 200 µL of 1% crystal violet (Sinopharm, Shanghai, China) stain was added to each well, and the plates were stained at room temperature for 15 min. After staining, the wells were washed three times with PBS to remove excess stains. Finally, 200 µL of 95% ethanol was added to dissolve the crystal violet from the biofilms. Absorbance at 570 nm was measured using a plate reader to quantify biofilm formation. For biofilm eradication, bacteria were seeded in a 96-well plate and incubated for 24 h to allow biofilm formation as described above. After incubation, the medium was removed, and the wells were washed three times with PBS to remove unattached cells. 100 µL of colistin or CH solution (at the same concentrations as above) and 100 µL of fresh medium were added to each well. The plates were incubated at 37 °C for another 24 h. After incubation, the wells were washed three times with PBS to remove loosely attached cells. 200 µL of 1% crystal violet was added to stain the biofilms, followed by three PBS washes. 200 µL of ethanol was added to dissolve the stained biofilms, and absorbance at 570 nm was measured to quantify the remaining biofilm mass. Each group was set up with five biological replicates. Additionally, SYTO 9 (5 µM) (Invitrogen, Carlsbad, CA, USA) was prepared and added to the wells, incubated in the dark at room temperature for 30 min, and the biofilms were visualized using confocal laser scanning microscopy. Live cells within the biofilm were stained green by the SYTO 9 dye. Each group was set up with three biological replicates.

### 4.8. Measurement of Outer Membrane Permeability Assay

The experimental procedures refer to the previous reference with minor modifications [43]. The fluorescent probe NPN (Beyotime, Shanghai, China) was used to assess the integrity of the outer membrane of *E. coli* treated with colistin or CH. In brief, bacteria were cultured overnight at 37 °C and 200 rpm, then transferred and grown to the logarithmic phase. The cultures were washed with PBS and resuspended. The OD600 of the bacterial suspension was adjusted to 0.5, and NPN dye was added to a final concentration of 10 µM. After incubation at 37 °C for 30 min, 190 µL of the dye-labeled bacterial suspension was added to a 96-well plate. Then, 10 µL of colistin or CH was added, and after 2 h of incubation, fluorescence was measured with an excitation wavelength of 350 nm and an emission wavelength of 420 nm. Each group was set up with three biological replicates.

### 4.9. Measurement of Inner Membrane Permeability Assay

The experimental procedures refer to the previous reference with minor modifications [44]. Bacteria in the logarithmic growth phase were washed and resuspended in PBS, and the OD600 was adjusted to 0.5. In the presence of colistin (0.125 μg/mL) or CH (8–32 μg/mL), 10 nM of PI dye (Beyotime, Shanghai, China) was added, and the bacteria were incubated at 37 °C for 2 h. After incubation, the excess dye was washed away with PBS, and the bacteria were transferred to black 96-well plates for fluorescence measurement. The excitation wavelength was 535 nm, and the emission wavelength was 615 nm. Each group was set up with three biological replicates.

### 4.10. ATP Content Detection

The experimental procedures refer to the previous reference with minor modifications [44]. Bacteria in the logarithmic growth phase were washed with PBS buffer and resuspended, adjusting the bacterial OD600 to 0.5. The bacterial suspension was distributed into a multi-well plate (96-well plate), with 100 µL of bacterial suspension per well. 100 µL of colistin and CH at various concentrations (individual and combination groups) were added, with a negative control group (no drug treatment). The final drug concentrations were based on previous experimental steps. The plates were incubated at 37 °C for 2 h to ensure sufficient drug action. After incubation, the bacterial suspension in the 96-well plate was centrifuged, and the supernatant was discarded. The bacterial pellet was washed three times with PBS to remove residual medium and drugs. An appropriate amount of lysis buffer (typically 100 µL/well) was added, and the bacterial pellet was gently agitated or pipetted to ensure complete lysis. The plate was left at room temperature for 10 min to ensure thorough lysis. After lysis, 100 µL of the lysate was transferred to a new 96-well plate. An equal amount of luciferase reagent (100 µL from the ATP detection kit) (Beyotime, Shanghai, China) was added and mixed in the dark. After incubation at room temperature for 5–10 min, fluorescence was measured using a luminometer, and intracellular ATP concentrations were calculated using an ATP standard curve. For extracellular ATP detection, the bacterial suspension was centrifuged after drug treatment, and the supernatant was collected for extracellular ATP measurement. 100 µL of the supernatant was transferred to a new 96-well plate, and ATP content was detected as described above for intracellular ATP. The fluorescence signal was read, and ATP concentration was calculated using an ATP standard curve. Each group was set up with three biological replicates.

### 4.11. Reactive Oxygen Species (ROS) Measurement

The experimental procedures refer to the previous reference with minor modifications [45]. Bacteria in the logarithmic growth phase were washed with PBS and resuspended, adjusting the OD600 to 0.5. 2′,7′-Dichlorodihydrofluorescein Diacetate (DCFH-DA) (Beyotime, Shanghai, China) was added to a final concentration of 10 µM, and the mixture was incubated at 37 °C for 30 min. The bacteria were washed three times with PBS and then treated with colistin or CH. The treated bacteria were incubated for 2 h, and the mixture was transferred to a black 96-well plate for fluorescence signal collection. Each group was set up with three biological replicates.

### 4.12. NO Detection

The experimental procedures refer to the previous reference with minor modifications [46]. Bacterial suspensions in the logarithmic growth phase were centrifuged and washed 2–3 times with PBS buffer, and the bacterial pellet was collected. The bacteria were resuspended in PBS buffer (OD600 = 0.5). Drugs were added, and the mixtures were incubated at 37 °C for 2 h. Samples were then collected, and nitric oxide levels were measured using a nitric oxide detection kit (Beyotime, Shanghai, China) according to the manufacturer’s instructions. Absorbance was measured at 540 nm for each sample. Each group was set up with three biological replicates.

### 4.13. Cytotoxicity Testing of Drug Combination

The experimental procedures refer to the previous reference with minor modifications [47]. Hemolysis assays were conducted using fresh sheep red blood cells (Yuanye, Shanghai, China). Fresh red blood cells were washed with PBS and centrifuged at 800 r/min at 4 °C for 10 min. A 10% red blood cell suspension was added to different concentrations of colistin or CH, with 1% Triton X-100 used as the positive hemolysis control and red blood cells in PBS as the negative control. The samples were incubated at 37 °C for 6 h, then centrifuged at 800 r/min for 10 min, and the supernatant was measured for absorbance at 540 nm. Each group was set up with three biological replicates.

Cytotoxicity on mammalian cells was tested on J774.A1 cells using the CCK8 (Beyotime, Shanghai, China) assay. Cells were cultured in DMEM containing 10% fetal bovine serum (FBS). After passage, the cells were seeded in 96-well plates. Cells were washed with PBS, and fresh medium containing different concentrations of colistin or CH was added, followed by 24-h incubation. The medium was then replaced with medium containing 10% CCK8, and after 22 h of incubation, absorbance at 590 nm was measured using a spectrophotometer. The survival rate was calculated using the formula: Cell viability = [(absorbance of experimental well − absorbance of blank well)/(absorbance of control well − absorbance of blank well)] × 100% [47]. Each group was set up with three biological replicates.

### 4.14. Galleria Mellonella Infection Model

The experimental procedures refer to the previous reference with minor modifications [48]. The therapeutic potential of colistin, CH, and the combination group was evaluated in a *Galleria mellonella* infection model. Larvae were randomly divided into four groups (n = 10 per group), and 10 μL of *E. coli* 42 suspension (1.0 × 10⁸ CFU/mL) was injected into the right rear proleg. One hour post-infection, the larvae were treated with colistin, CH, or the combination group by injecting into the left rear proleg. Each larva was injected with 10 uL of drug solution, and PBS buffer was used as solvent control. Larvae survival was monitored for 5 days, with dead larvae turning black.

### 4.15. Mouse Infection Model

The experimental procedures refer to the previous reference with minor modifications [48]. To evaluate the therapeutic efficacy of colistin and CH in an animal model of bacterial infection, female SPF-grade BALB/c mice were purchased from Huazhong Agricultural University. Six or eight-week-old female BALB/c mice weighing 16 ± 2 g (n = 10 per group) were randomly divided into six groups. The mice were infected intraperitoneally with 3.5 × 10⁸ CFU of *E. coli* 42 suspension. One hour post-infection, colistin, CH, or the combination group was administered via gavage, and each mouse was administered 100 uL of drug solution, and ddH_2_O buffer was used as solvent control. Survival was recorded over 7 days. For the non-lethal mouse model, six or eight-week-old female BALB/c mice weighing 16 ± 2 g (n = 5 per group) were randomly divided into five groups and infected intraperitoneally with 1 × 10⁸ CFU of *E. coli* 42 suspension. The treatment procedure was the same as above. Twelve hours after treatment, mice were anesthetized, and cardiac blood was collected to measure serum levels of IL-6 and TNF-α. The liver and spleen were homogenized and serially diluted, then plated on LA plates. The samples were incubated at 37 °C overnight, and bacterial counts were performed. Portions of lung tissue were fixed in 4% paraformaldehyde for pathological analysis.

### 4.16. Statistical Analysis

Statistical analysis of independent samples was performed using GraphPad Prism 8.0. Experimental results were analyzed using either a two-tailed unpaired *t*-test or one-way ANOVA. All data collected from at least triplicates were presented as means ± SD. A *p*-value of * *p* < 0.05 was considered statistically significant, ** *p* < 0.01 as highly significant, *** *p* < 0.001 (**** *p* < 0.0001) as extremely significant, and “ns” indicates no significant difference.

## 5. Conclusions

In conclusion, this study showed that the combination of colistin and CH significantly restored colistin sensitivity in *mcr-1*-positive *E. coli*, revealing their synergistic antibacterial mechanism involving membrane damage and oxidative stress, with promising clinical applications. Future research could further explore the mechanism of combination therapy and conduct large-scale clinical trials, offering new solutions for treating infections caused by multidrug-resistant bacteria.

## Figures and Tables

**Table 1:** Test results of seven drugs in combination with colistin E.

Drugs	*E. coli* 42		
	MIC of COL	MIC of COL with Drugs (32 µg/mL)	MIC Fold Change of COL
Oxiconazole nitrate	4	0.0625	64
Diethylstilbestrol	4	0.25	16
Guanfacine hydrochloride	8	4	2
Sulfabenzamide	4	4	1
Cinacalcet hydrochloride	8	≤0.0625	≥64
Pramipexole	4	4	1
Sorafenib	4	0.125	32

## Data Availability

The data presented in this study are available on request from the corresponding author.

## Supplementary Materials

The following supporting information can be downloaded at: https://www.mdpi.com/article/10.3390/ijms252111574/s1.
