# Supplementary material for: Restoring Colistin Sensitivity in Multidrug-Resistant Pathogenic E. coli Using Cinacalcet Hydrochloride
Source: Int J Mol Sci. 2024 Oct 28;25(21):11574. doi: 10.3390/ijms252111574 (PMC11546906; doi:10.3390/ijms252111574)
Supplement: Supplementary file 1 [file ijms-25-11574-s001.zip › Supplementary Materials.pdf]

Table. S1 MICs of clinical antibiotics in tested bacterial strains.

	MCR-1 confirmation	MIC(μg/mL)							
		LEV	DOX	GEN	AMP	CEF	CL	COL	CH
E. coli ATCC 25922	-	＜0.015	0.5	2	4	<0.125	2	0.5	32
E. coli 1347	-	16	16	128	>1024	>256	>256	0.0625	256
E. coli 1808106	-	8	32	1	>1024	0.25	64	0.5	>256
E. coli 1351	-	0.5	32	1	>1024	>256	16	0.0625	64
E. coli 17039	-	8	32	>128	>1024	<0.125	128	0.03125	256
E. coli 1240	-	>32	16	>128	>1024	256	256	0.25	256
E. coli 19097	+	4	8	8	128	8	4	4	256
E. coli 1244	+	32	32	128	>1024	128	64	2	128
E. coli 1704087	+	4	16	2	>1024	256	256	8	64
E. coli 1203	+	8	32	128	>1024	256	128	2	256
E. coli 16867	+	>32	16	>128	>1024	256	128	4	128
E. coli 42	+	16	32	128	>1024	128	256	4	>256

LEV, Levofloxacin; Doxycycline, DOX;Gentamicin, GEN; Ampicillin, AMP;CEF, Cefotaxime Sodium;CL,Chloramphenicol;COL, Colistin E; CH, Cinacalcet hydrochloride;

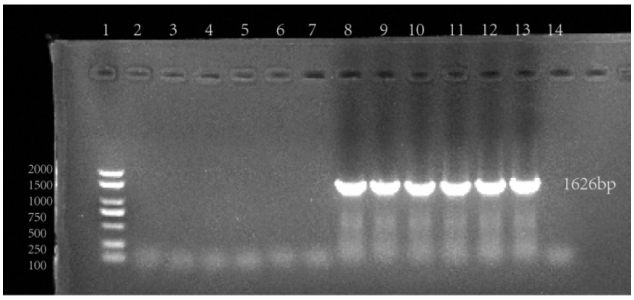

Figure S1. Amplification of the *mcr-1* gene in 11 *E. coli* strains. 1, mark; 2, *E. coli* ATCC 25922; 3, *E. coli* 1347; 4, *E. coli* 1808106; 5, *E. coli* 1351;6, *E. coli* 17039; 7, *E. coli* 1240; 8, E. coli 19097; 9, *E. coli* 1244; 10, *E. coli* 1704087; 11, *E. coli* 1203; 12, *E. coli* 16867; 13, *E. coli* 42; 14, ddH2O.

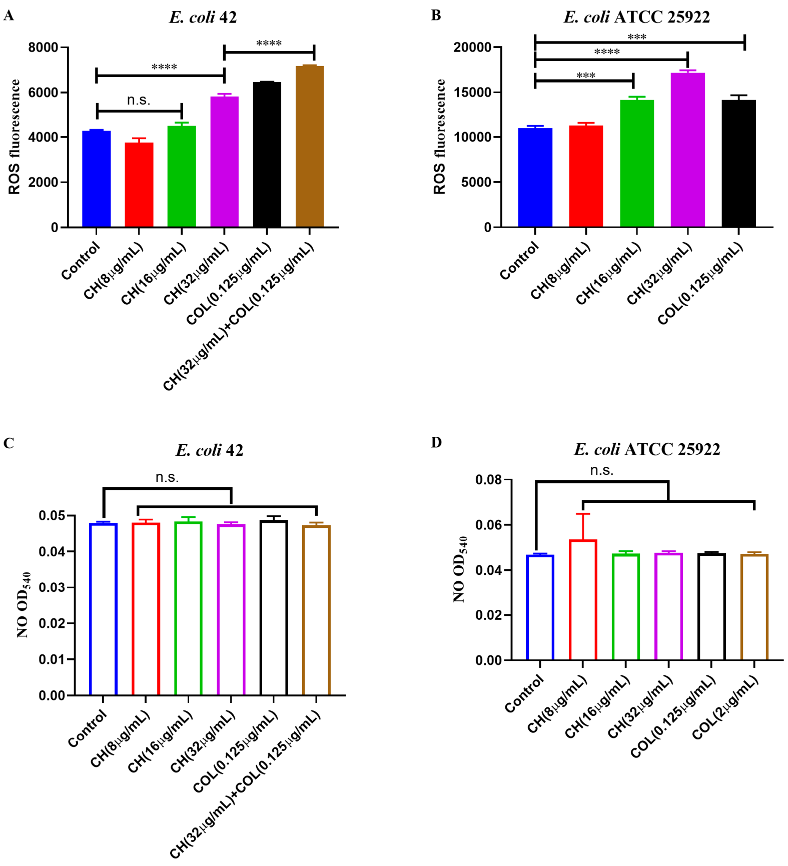

Figure S2. Effect of CH combined with colistin on the bacterial ROS and NO. The bacterial NO production were determined using Nitric Oxide assay kit (A, B);The bacterial ROS production were determined. probed with 2’,7’-dichlorodihydrofluorescein diacetate (DCFH-DA)(C, D). Data were mean ± SEM for n = 3 biologically independent experiment. n.s. represents no significance, *** p < 0.001, ****p < 0.0001 vs control.
